# 983. Pathogen-specific PET/CT imaging for Gram negative implant-associated spinal infections

**DOI:** 10.1093/ofid/ofac492.825

**Published:** 2022-12-15

**Authors:** Oren Gordon, Anna Napiorkowski, Kelly Flavahan, Trisha De Jesus, Dustin Dikeman, Amy Kronenberg, Nathan Archer, Sanjay K Jain

**Affiliations:** Hadassah-Hebrew University Medical Center, Jerusalem , Israel; Johns Hopkins, Baltimore, Maryland; Johns Hopkins, Baltimore, Maryland; Johns Hopkins, Baltimore, Maryland; Johns Hopkins, Baltimore, Maryland; Johns Hopkins, Baltimore, Maryland; Johns Hopkins, Baltimore, Maryland; Johns Hopkins, Baltimore, Maryland

## Abstract

**Background:**

Rapid and accurate diagnosis of bacterial implant-associated spinal infection is essential for early intervention, surgical planning and rational use of antibiotics. While this infection is predominantly caused by Gram positive bacteria, some patients are at high risk for intestinal-derived Gram negative bacteria. However, current diagnostic tools require invasive sampling with substantial risks to the patient. Moreover, conventional imaging, including computed tomography (CT), magnetic resonance imaging (MRI) and positron emission tomography (PET) with 2-deoxy-2-[^18^F]fluoro-D-glucose (^18^F-FDG), cannot differentiate infection from non-infectious processes.

**Methods:**

PET/CT with 2-deoxy-2-[^18^F]fluoro-D-sorbitol (^18^F-FDS) is the first imaging modality specific for a bacterial class. ^18^F-FDS accumulates selectively in *Enterobacterales*, but not in Gram positive bacteria or mammalian cells and was safe in phase I-II clinical studies. Here, we developed a spinal infection model utilizing a previously described posterior-approach to implant a titanium Kirschner wire into the L4 spinous process in mice. We compared ^18^F-FDS with ^18^F-FDG PET/CT in mice with spinal implants infected with *Staphylococcus aureus* (Figure 1), *Escherichia coli* (Figure 2) or without infection (but with post-surgical inflammation).

**Figure 1.**
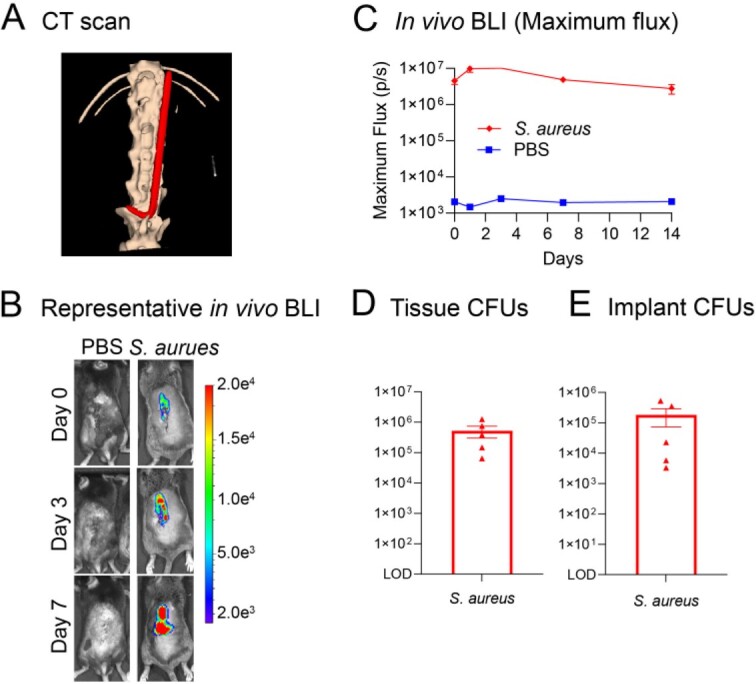
A mouse model of Gram-positive implant-associated spinal infection.

Surgical technique was adapted from Dworky et al., (J Orthop Res. 35, 193-199. 2017). Briefly, a 2 cm midline incision was made in the skin and the L4 spinous process was exposed. An orthopedic-grade titanium Kirschner wire (diameter 0.1 mm) was surgically placed into the L4 spinous process and placed lengthwise along the spine. A bioluminescent S. aureus (SAP231; 1 x 106 colony forming unites [CFUs]) in 10 μL) or phosphate-buffered saline (PBS) were pipetted onto the implant. The model was performed with S. aureus (n=5 mice) or PBS (n=1 mouse) and mice were followed for 14 days for in vivo bioluminescent imaging (BLI) before they were sacrificed for ex vivo CFU enumeration. (A) Computed tomography (CT) of the mouse spine indicating the implant in red. (B) Representative in vivo S. aureus BLI signals on a color scale overlaid on top of a grayscale image of the backs of the mice. (C) Mean in vivo BLI (Maximum flux, photons / second ± s.e.m (logarithmic scale). (D-E) On post-operative day 14 the implants were removed and sonicated, and the infected vertebra with surrounding soft tissue were harvested and homogenized. Mean ex vivo CFUs ± s.e.m from tissue and implants are shown (n=5).

**Figure 2.**
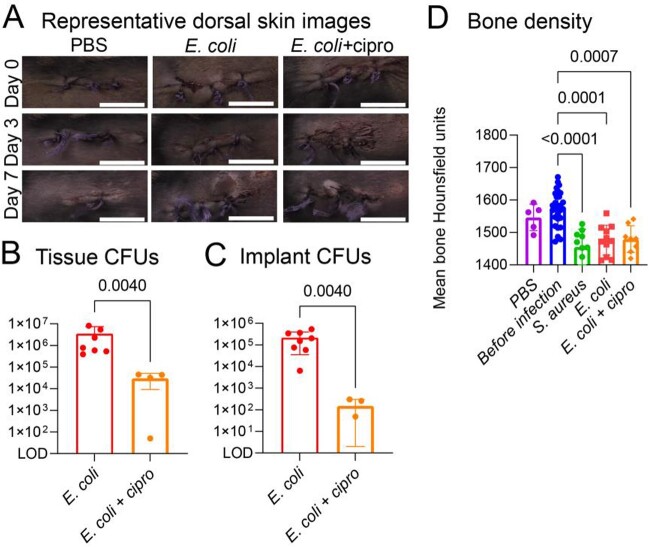
A mouse model of Gram-negative implant-associated spinal infection.

Similar surgical technique was used as before (Figure 1) and the model was performed with E. coli (ATTC-25922; 1 x 106 CFUs; n=8 mice) or PBS (n=3 mouse). Mice were followed for 14 days before they were sacrificed for ex vivo CFU enumeration. An additional group of mice infected with E. coli (n=4) received antibiotic treatment (Intraperitoneal [IP] ciprofloxacin 20mg/kg/dose twice a day for 5 days) before sacrifice. (A) Representative dorsal skin images showing the surgical site. Note the development of skin wounds around the surgical site in the E. coli infected, but not the treated mice. Scale bars: 1 cm. (B-C) On post-operative day 14 the implants were removed and sonicated, and the infected vertebra with surrounding soft tissue were harvested and homogenized. Mean ex vivo CFUs ± s.e.m from tissue and implants are shown. (D) Bone remodeling was evaluated using μCT to measure bone density in Hounsfield units (HU). The mean HU of bone (defined as HU>700) is presented for mice with PBS (n=3 mice and 5 scans), mice imaged on day of surgery (before infection; n=15 mice and 29 scans), S. aureus (n=5 mice, 10 scans), E. coli (n=6 mice, 11 scans) and E. coli treated with ciprofloxacin (n=4 mice, 8 scans). Note the lower HU for infected animals (S. aureus or E. coli) compared to non-infected (PBS or before infection). Five days of treatment with ciprofloxacin were not sufficient to mitigate bone remodeling in this model. P values are indicated as shown by the 2-tailed Mann-Whitney test (B-C) or the Kruskal-Wallis test adjusted for multiple comparisons to preserve the desired false discovery rate (D).

**Results:**

Both bacteria induced substantial bone pathology with reduced bone density (Figure 2D). ^18^F-FDS PET/CT could specifically detect implant-associated spinal infections due to *E. coli* with mean target-to-non-target standard uptake value (SUV) ratio of 9.2 ± 1.5, which was substantially lower in *S. aureus* (3.0 ± 0.4) and uninfected mice (4.3 ± 0.3; *P*=0.002; Figure 3). In contrast, ^18^F-FDG could not differentiate between the two bacterial infections or the controls (*P*=0.497; Figure 4). Finally, ^18^F-FDS monitored the efficacy of antibiotic treatment, demonstrating a signal proportional to bacterial burden (Figure 5).

**Figure 3.**
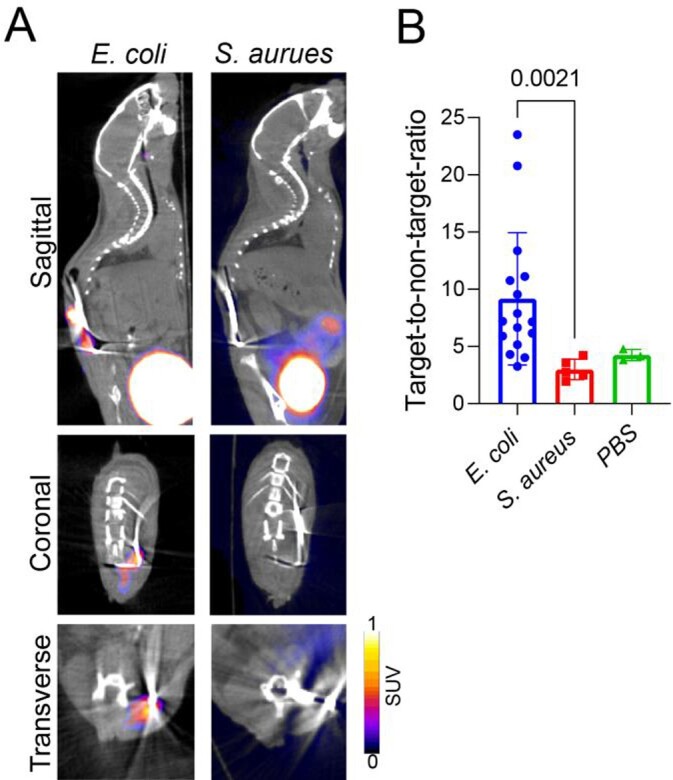
18F-FDS PET/CT imaging for detection of Gram-negative implant associated spinal infection in mice.

The mouse model was performed with either E. coli (n=15), S. aureus (n=5) or PBS (n=3) and mice were imaged with 18F-FDS PET/CT imaging. (A) Representative images of 18F-FDS PET/CT. (B) Regions of interest (ROIs) were drawn around the implant (Infected target) and in the heart (uninfected non-target). Standard uptake values (SUVs) are presented as ratios between infected and non-infected ROIs. P values are indicated as shown by the Kruskal-Wallis test adjusted for multiple comparisons to preserve the desired false discovery rate.

**Figure 4.**
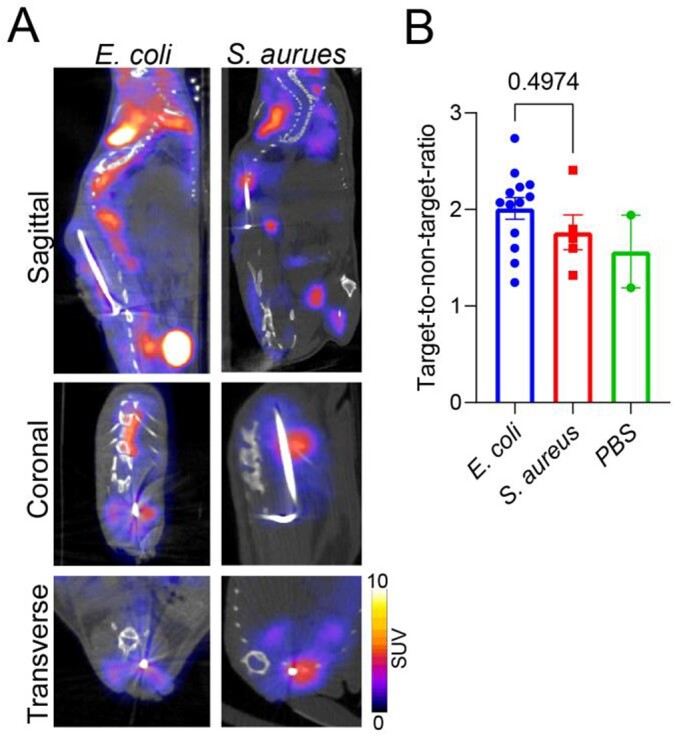
18F-FDG PET/CT imaging for implant associated spinal infection in mice.

Same mice used for 18F-FDS imaging (Figure 3) were imaged with 18F-FDG PET/CT as follows: E. coli (n=13), S. aureus (n=5) or PBS (n=2). (A) Representative images of 18F-FDG PET/CT. (B) Regions of interest (ROIs) were drawn around the implant (Infected target) and in the liver (uninfected non-target). Standard uptake values (SUVs) are presented as ratios between infected and non-infected ROIs. P values are indicated as shown by the Kruskal-Wallis test adjusted for multiple comparisons to preserve the desired false discovery rate.

**Figure 5.**
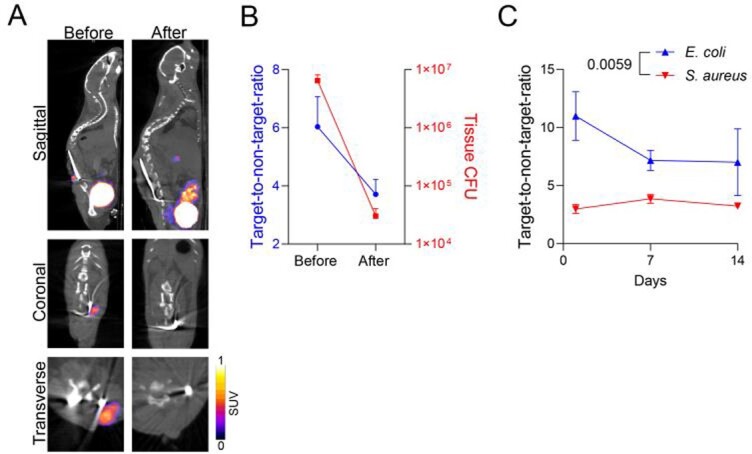
18F-FDS PET/CT imaging to monitor infection over time and following antibiotic treatment.

The mouse implant-associated spinal infection model was performed with E. coli (n=4) and mice were imaged with 18F-FDS PET/CT before and after completion of antibiotic treatment (IP ciprofloxacin 20mg/kg/dose every 12 hours for 5 days). One day later, mice were sacrificed and the infected vertebra with surrounding soft tissue were harvested and homogenized for ex vivo enumeration of CFUs. (A) Representative images of 18F-FDS PET/CT. (B) ROIs were drawn around the implant (Infected target) and in the heart (uninfected non-target). SUVs are presented as ratios between infected and non-infected ROIs and mean ex vivo CFUs ± s.e.m from tissue are shown, before and after antibiotic treatment. P = 0.014 for the difference in CFU counts and P = 0.032 for the difference in SUV ratios, before and after antibiotic treatment as shown by the one-tailed Mann-Whitney test. (C) The mouse implant-associated spine infection model was performed with E. coli or S. aureus and mice were imaged with 18F-FDS PET/CT at days 1, 7 or 14 following infection (n=4-10 mice/time point). P value is indicated as shown by the two-way analysis of variance (ANOVA) test.

**Conclusion:**

In this preclinical study, ^18^F-FDS PET/CT rapidly and specifically detected *E.coli* implant-associated spinal infection. We are currently conducting a clinical study to evaluate ^18^F-FDS PET/CT for specific detection of *Enterobacterales* implant-associated infection which may reduce the need for surgical interventions.

**Disclosures:**

**Nathan Archer, PhD**, Janssen: Advisor/Consultant|Pfizer: Grant/Research Support **Sanjay K. Jain, MD**, Fujirebio Diagnostics, Inc., USA: Grant/Research Support|Novobiotic LLC, USA: Grant/Research Support|T3 Pharma, Switzerland: Grant/Research Support.

